# The study of metabolic improvement by nutritional intervention controlling endogenous GIP (Mini Egg study): a randomized, cross-over study

**DOI:** 10.1186/s12937-019-0472-0

**Published:** 2019-09-02

**Authors:** Naoki Sakane, Noriko Osaki, Hideto Takase, Junko Suzuki, Chika Suzukamo, Shinsuke Nirengi, Akiko Suganuma, Akira Shimotoyodome

**Affiliations:** 1 Division of Preventive Medicine, Clinical Research Institute, National Hospital Organization Kyoto Medical Center, 1-1 Mukaihata-cho, Fukakusa, Fushimi-ku, Kyoto, 612-8555 Japan; 2 Biological ScienceResearch, Kao Corporation, 2-1-3 Bunka, Sumida, Tokyo, 131-8501 Japan; 3 Biological Science Research, Kao Corporation, 2606 Akabane, Ichikai-machi, Haga-gun, Tochigi, 321-3497 Japan

**Keywords:** Glucose-dependent insulinotropic polypeptide, Visceral fat, Traditional Japanese diet

## Abstract

**Background:**

Given the major role of glucose-dependent insulinotropic polypeptide (GIP) in the regulation of adiposity, this study examined the effects induced by a diet based on the Japanese tradition (SMART WASHOKU) on the visceral fat area (VFA) and GIP secretions.

**Methods:**

Overweight/obese men (*n* = 21; mean age, 41.0 ± 9.0 years; mean BMI, 25.2 ± 2.0 kg/m^2^) without diabetes were placed on either a SMART WASHOKU or control meal for 2 weeks, in a randomized, cross-over setup with a four-week washout period.

**Results:**

For the meal tolerance test, blood samples were collected at 0, 30, 60, 120, 180, and 240 min post-meal, followed by measuring blood glucose, insulin, GIP, and glucagon-like peptide-1 (GLP-1) levels. Relative to a control meal, SMART WASHOKU meal yielded significantly lower plasma postprandial GIP concentrations (AUC: 700.0 ± 208.0 vs. 1117.0 ± 351.4 pmol/L・4 h, *P* < 0.05); however, between meals, there was no significant difference in the levels of GLP-1, peptide YY, and ghrelin. Compared to the control meal, SMART WASHOKU intervention significantly reduced VFA and the levels of LDL-cholesterol, triglyceride, and HbA1c after the chronic meal intervention.

**Conclusions:**

In conclusion, a SMART WASHOKU meal may decrease VFA and improve metabolic parameters in overweight/obese men, possibly via suppressing GIP secretion.

## Background

Glucose-dependent insulinotropic polypeptide (GIP) is an incretin hormone produced by K cells in the upper gastrointestinal tract [1]. The action of GIP is mediated by interactions with the GIP receptor that induces pancreatic beta-cells to release insulin [1]. In addition to pancreatic beta-cells, adipocytes express functional GIP receptors [2]. Animal studies have demonstrated that the regulation of adiposity is an important physiological function of GIP [3]. GIP induces glucose uptake, the activity of lipoprotein lipase, and induction of triglycerides by 3 T3-L1 adipocytes [4]. Deletion of GIP receptor counteracts diet-induced obesity in leptin-deficient mice, and conversely, administration of a GIP receptor antagonist was found to suppress weight gain in mice fed with a high-fat diet [5]. In a cross-sectional study, GIP secretion in early phase was found to be positively correlated with body mass index (BMI) in non-obese and obese patients with type 2 diabetes mellitus [6]. Common variants of GIP are known to be associated with the accumulation of visceral fat in humans [7, 8]. Therefore, GIP is considered to play a critical role in the regulation of adiposity, especially visceral fat accumulation. Diet based on the Japanese tradition (A WASHOKU) is characterized by high consumption of fish and soybean products and low consumption of animal fat and meat [9]. Previously, the WASHOKU dietary pattern was shown to be associated with a decreased risk of cardiovascular mortality [10–13]. In a cross-sectional study, we found that a protein/fat ratio ≈1.0, dietary fiber/carbohydrate ratio (≧ 0.063), and n-3 fatty acid/fat ratio (≧ 0.054) were negatively associated with the accumulation of visceral fat [14]. Therefore, SMART WASHOKU was defined as Japanese dietary patterns with a protein/fat ratio ≈1.0, dietary fiber/carbohydrate ratio ≥ 0.063, and n-3 fatty acid/fat ratio ≥ 0.04. However, it remained unknown whether the SMART WASHOKU dietary intervention decreases visceral fat area (VFA); further, the mechanism associated with the reduced VFA have not been described. The K-cell is considered to directly sense and respond to numerous nutrients in the intestine [15]. GIP secretions were suppressed by a vegetable-rich mixed meal [16], although a high-fat diet enhanced GIP secretion [17]. Therefore, SMART WASHOKU might suppress GIP secretions. We hypothesized that a SMART WASHOKU suppressed GIP secretions after meals and decreased VFA in overweight/obese males. The present study aimed to examine and compare the effects of SMART WASHOKU and modern Japanese meals on VFA and GIP secretions in overweight/obese men without diabetes.

## Methods

### Trial design

**Fig. 1:**
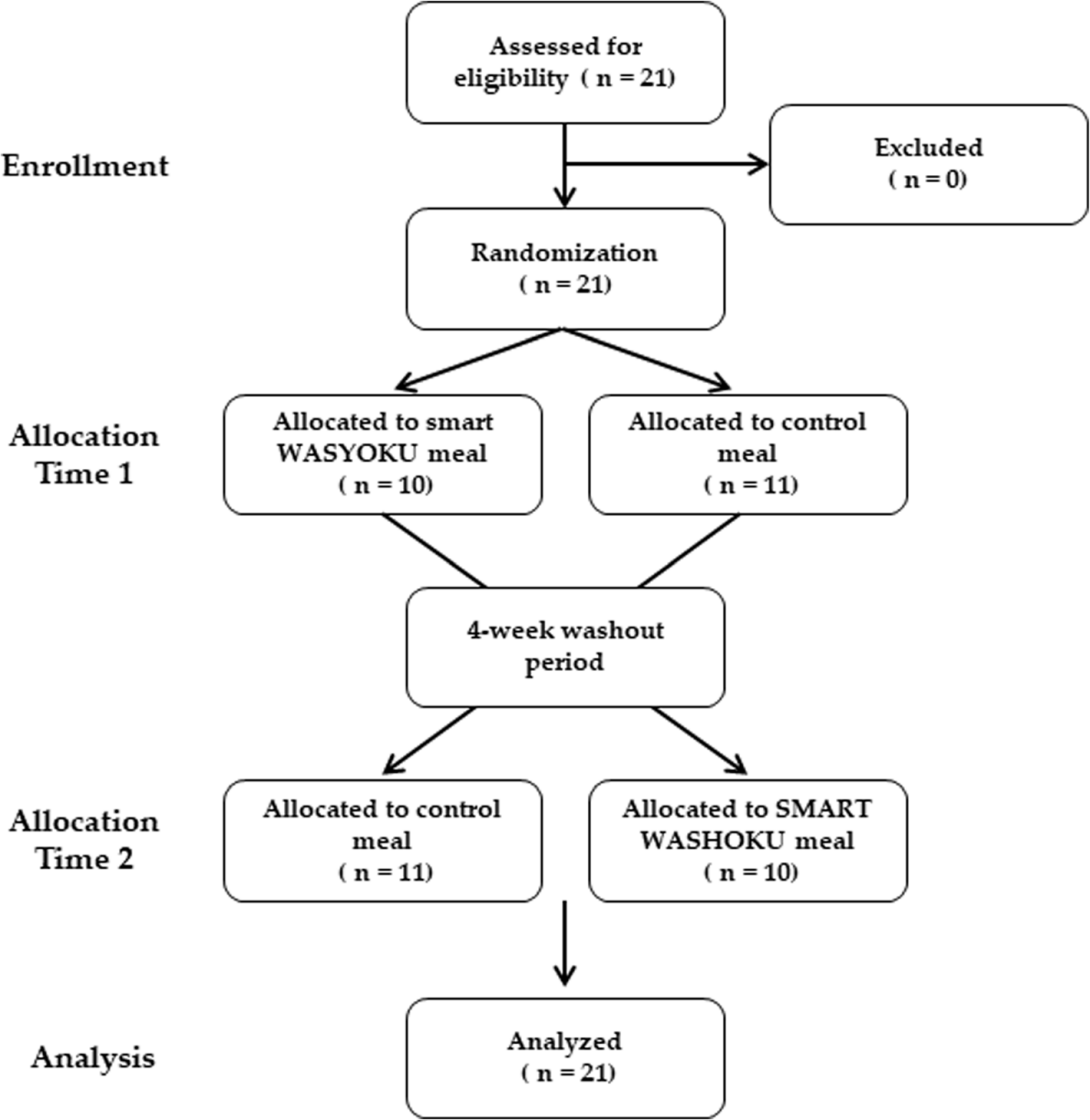
CONSORT flow diagram. This figure shows the flow of participants through the trial according to the criteria recommended in the CONSORT Guidelines

This study was a single-center, randomized, two-way crossover trial. Enrolled participants were overweight and obese men without diabetes. The study consisted of two intervention periods of 2 weeks separated by a washout period of 4 weeks (Fig. 1). Before the chronic meal intervention trial, a meal tolerance test was performed. The meal tolerance test was a randomized, two-way crossover trial with a washout period of 1 week. All subjects gave their informed consent for their participation in this study. The study was performed in accordance with the Declaration of Helsinki, approved by the Institutional Review Board of Kyoto Medical Center (2014/12/25), and registered at University hospital Medical Information Network (UMIN) center (UMIN000016772).

### Participants and their selection

Twenty-one individuals were recruited through poster advertisement and word of mouth across Kao Corporation. Following inclusion criteria were applied during the selection of candidates: (1) Japanese males between 20 and 59 years of age; (2) BMI ≥ 23 kg/m^2^; (3) informed consent provided; (4) able to receive and cook experimental diet; (5) able to follow the instructions of the study team; and (6) participated in a meal tolerance test. The exclusion criteria were as follows: (1) plasma glucose value ≥126 mg/dL (7.0 mmol/L), LDL-cholesterol value ≥180 mg/dL (4.65 mmol/L), triglyceride value ≥300 mg/dL (3.4 mmol/L), systolic blood pressure ≥ 160 mmHg; (2) weight change during the past year ≥3 kg; (3) severe liver or renal dysfunctions; (4) shift and night-shift workers; (5) food allergy and suspected food allergy (self-reported); (6) an unbalanced diet (not able to eat fish and meat); (7) poor physical health such as rapid weight loss during the past 2 months; (8) plan to lose weight through vigorous exercise and resistance training; and (9) subjects determined ineligible (for some other reasons) by the investigator/project leader. Sex-based differences in the body fat distribution were observed [18, 19]. Males are more likely to accumulate visceral fat than females. Therefore, we selected males only in this study. The cut-off for BMI was according to WHO standard criteria (underweight < 18.5, normal 18.5–24.9, overweight ≥25–29.9, and obese ≥30 kg/m^2^) [20, 21]. Asian populations have a lower BMI associated with increased health risks compared with Caucasian populations [22]. Therefore, when this is taken into account, the cut-off of Asia-Pacific BMI was set as ≥23 for overweight according to the Regional Office for the Western Pacific Region of the WHO [23, 24]. The study team adopted BMI of ≥23 for potential health action points. This study did not include subjects who took diabetes medications.

### Meal tolerance test

There were two different test meals, SMART WASHOKU and control. The test meals consisted of staple food, main dish, side dish, and soup. The energy compositions of the test meals are shown in Table 1.

**Table 1:** Test meal composition of the Smart WASHOKU and control meals

Variables	Smart WASHOKU	Control meal
Test meal		
Staple food	Steamed brown rice	Small portion steamed rice
Main dish	Simmered mackerel with soy sauce	Hashed beef
Side dish	Simmered daikon, fried fish balls and shiitake mushrooms in soy sauce, simmered vegetables and beans,	Boiled quail eggs, processed cheese
Soup	Wakame seaweed soup	-
Total energy, kcal	661	636
Protein, %	20.5	14.2
Fat, %	22.6	34.4
Carbohydrate, %	56.9	51.5
Protein/fat ratio	0.905	0.412
Fiber/carbohydrate ratio	0.154	0.027
n-3 fatty acid/lipid ratio	0.090	0.019

During the day before each experimental day, subjects were encouraged to standardize their meal pattern and maintain regular eating habits. They were also instructed to avoid alcohol and excessive physical exercise during the day prior to the experimental day. On the experimental days, a test meal was served at 9:00 am, and the participants were required to finish their meal within 15 min. Blood samples were collected at the following time points (minutes) before breakfast: 0, 30, 60, 120, 180, and 240.

### Intervention

The study consisted of two periods of 2 weeks separated by a washout period of 4 weeks. SMART WASHOKU meals contained 2096 kcal energy, 22.3% protein, 20.8% fat, dietary fiber/carbohydrate ratio ≥ 0.063, and n-3 fatty acids/fat ratio ≥ 0.04. Control meals contained 2096 kcal energy, 13.7% protein, 37.5% fat, dietary fiber/carbohydrate ratio ≤ 0.063, n-3 fatty acids/fat ratio ≤ 0.04. For 14 consecutive days, the participants were provided with commercially available meals (Table 2). We provided one meal selected from 5 meals for breakfast or lunch, and one meal selected from 5 meals for dinner by the rotation method. This is an example of the experimental diet (Table 2). The test meals were provided at work sites on a working day. If participants requested meals at home, we delivered them to the homes. On holidays, we also delivered meals to their homes. Dietary adherence was based on daily nutrition logs. It was measured as the percentage of meals provided by the research group that participants ate.

**Table 2:** Example of experimental diet (Wednesday)

Variables	Smart WASHOKU	Control meal
Breakfast		
Staple food	Steamed brown rice	Small portion steamed rice
Main dish	Simmered mackerel with soy sauce	Hashed beef
Side dish	Simmered daikon, fried fish balls and shiitake mushrooms in soy sauce, simmered vegetables and beans,	Boiled quail eggs, processed cheese
Soup	Wakame seaweed soup	-
Lunch		
Staple food	Steamed brown rice	Beef rice bowl
Main dish	Japanese amberjack teriyaki	
Side dish	Simmered bamboo shoots, butterbur and fried tofu in soy sauce, Simmered beans	Pumpkin salada, boiled quail eggs
Soup	Miso soup with welsh onion	-
Dinner		
Staple food	Steamed rice with mixed grains	Steamed rice
Main dish	Salt-grilled salmon	Grilled pork with ginger
Side dish	Simmered vegetable and beans, simmered pumpkin, simmered hijiki seaweed, simmered daikon, fried fish balls and shiitake mushrooms in soy sauce	Potato salada,Processed cheese
Soup	Miso soup with wakame seaweed	-

### Outcomes

The primary endpoint was the measurement of VFA by the abdominal bioelectrical impedance analysis (EW-FA90, Panasonic) [25]. VFA based on abdominal bioelectrical impedance analysis was correlated significantly with VFA determined by abdominal computed tomography (*r* = 0.88). The coefficient of variation of test-retest reliability was 0.89%. Additional endpoints included determining weight, waist circumference, blood pressure, serum lipid, and GIP levels. We used a digital anthropometric scale (with an accuracy of 0.1 kg) for the evaluation of body weight (kg), and a stadiometer (with an accuracy of 0.1 cm) to obtain height (m) of the participants. BMI was calculated by the ratio of weight (kg) by the square of height (m). Asian-specific BMI cut-offs were used to define overweight (23.0 to < 27.5 kg/m^2^) and obese (≥ 27.5 kg/m^2^) [26].

The following variables were measured: Blood glucose (ACCU-CHEK AVIVA, Roche Diagnostics K.K.); serum insulin (enzyme linked immunoassay (ELISA) kits, Mercodia Human Insulin ELISA Kit Mercodia AB); serum GIP (Human GIP (Total) ELISA Kit, EMD Millipore Co.); serum GLP-1 (Glucagon-Like Peptide-1(Active) ELISA Kit, EMD Millipore Co., Billerica, MA, USA); serum ghrelin (active) pancreatic polypeptide (MILLIPLEX MAP Human Metabolic Hormone Panel, EMD Millipore Co.); serum peptide (Human PYY (Total) ELISA Kit, EMD Millipore Co, Billerica, MA, USA). All assays had the inter-assay coefficient of variation (CV) or intra-assay CV of < 10%. HOMA-IR and HOMA-β values were calculated to determine the insulin resistance and functional capacity of pancreatic beta cells, respectively [27]. Serum lipids, liver enzyme, and high sensitivity C-reactive protein (hs-CRP) were also measured. HbA1c levels were measured by the enzymatic method.

We measured the dietary status before dietary intervention and during the washout period. Dietary assessment was performed using a brief, validated self-administered diet-history questionnaire [28].

### Sample size

Sample sizes were estimated using the xsampsi command, which is Stata module to calculate the sample size for cross-over trials with continuous measures. It was calculated with the assumption that an improvement of 10 cm^2^ in VFA was clinically relevant. With an expected standard deviation (SD) within measurements of VFA of 15, alpha of 0.05, and statistical power of 0.8, we needed to study 18 participants in a cross-over design. However, considering the risk of drop-out, we choose to include 20 participants.

### Randomization

The biostatistician supervisor, who was not aware of participants’ conditions, randomly assigned patients (1:1) to either type A meal during period 1 followed by type B meal during period 2 (AB sequence); further, the reverse order of the meals (BA sequence) was also followed. Random allocation was performed with randomization software using stratification based on age group (< 40 or ≥ 40 years) and BMI (< 25 or ≥ 25 kg/m^2^).

### Blinding

Study participants and investigators were not blinded to the dietary regimen. Analysts who performed final data analysis were blinded.

### Statistical analysis

All the data were disclosed after the termination of the trial and analyzed by an independent investigator. Data are reported as mean (SD). The postprandial variations were integrated as the area under the curve (AUC). For each measurement, AUC was calculated according to the trapezoidal rule. Data for different time-points and treatments were analyzed by repeated-measures ANOVA, usingtwo factors (time; and time and treatment) as factors. The data were analyzed by analysis of variance using the pkcross command in Stata/IC 13.1 software. The *P*-value was calculated using the pkcross command in Stata/IC 13.1 software because of the cross-over design. When analyzing trial data with the pkcross command, if the treatment, carryover, and sequence variables are known, the omnibus test for separability of the treatment and carryover effects is calculated. A *p* value of < 0.05 was considered to be significantly different.

### Adverse event assessment

Safety was assessed by the number of participants with adverse events (AEs). Using terms from the Medical Dictionary for Regulatory Activities, version 11.1, AE data were collected by systematic assessment of the participants who received one or more doses of intervention. AEs were not documented for the washout phase of the study.

## Results

### Recruitment and participants’ characteristics

A total of 21 participants were included in the current study (Table 3), and all of them completed the study successfully (Fig. 1). Adherence with the diet regimen was 96 ± 7% and 93 ± 10% in a SMART WASHOKU and control meal, respectively. Baseline total energy, protein, fat, carbohydrate, and fiber intakes in participants were 2003 ± 395 kcal, 15.3 ± 2.2%, 31.4 ± 5.8%, 53.2 ± 6.6%, and 14.0 ± 5.6 g, respectively. The baseline protein/fat ratio, dietary fiber/carbohydrate ratio, and n-3 fatty acids/fat ratio were 0.50 ± 0.10, 0.058 ± 0.020, and 0.035 ± 0.012, respectively. These dietary patterns in participants were similar to control diets. The protein/fat ratio, dietary fiber/carbohydrate ratio, and n-3 fatty acid/fat ratio during the washout period were 0.55 ± 0.13, 0.059 ± 0.017, and 0.036 ± 0.014, respectively. These dietary patterns during the washout period in participants were similar to baseline dietary patterns. There was no difference in total calorie intake between a SMART WASHOKU and the control diet (2097 ± 42 and 2110 ± 20 kcal, respectively). The protein/fat ratio, dietary fiber/carbohydrate ratio, and n-3 fatty acid/fat ratio during the intervention of a SMART WASHOKU were 1.09 ± 0.11, 0.118 ± 0.014, and 0.125 ± 0.019, respectively. The protein/fat ratio, dietary fiber/carbohydrate ratio, and n-3 fatty acid/fat ratio during the intervention of the control diet were 0.37 ± 0.03, 0.041 ± 0.005, and 0.023 ± 0.003, respectively.

**Table 3:** Baseline characteristics of participants

Variables	Mean (SD)
Age, years	41.0 (9.0)
Body weight, kg	73.1 (8.2)
BMI, kg/m^2^	25.2 (2.0)
Body fat, %	25.5 (4.7)
VFA, cm^2^	107.7 (30.6)
Waist circumference, cm	90.1 (4.7)
Hip circumference, cm	99.4 (4.4)
Fasting plasma glucose, mg/dL	94.4 (21.2)
HbA1c, %	5.5 (0.5)
Triglyceride, mg/dL	125.4 (58.2)
LDL-cholesterol, mg/dL	119.7 (25.8)
HDL-cholesterol, mg/dL	56.1 (13.5)
Systolic blood pressure, mmHg	131.5 (13.1)
Diastolic blood pressure, mmHg	87.5 (11.4)

^1^Data are presented as mean (SD). *BMI* Body mass index, *VFA* Visceral fat area, *HbA1c* Hemoglobin A1c, *LDL-cholesterol* Low-density lipoprotein cholesterol, *HDL-cholesterol* High-density lipoprotein cholesterol

### Meal tolerance test results

**Fig. 2:**
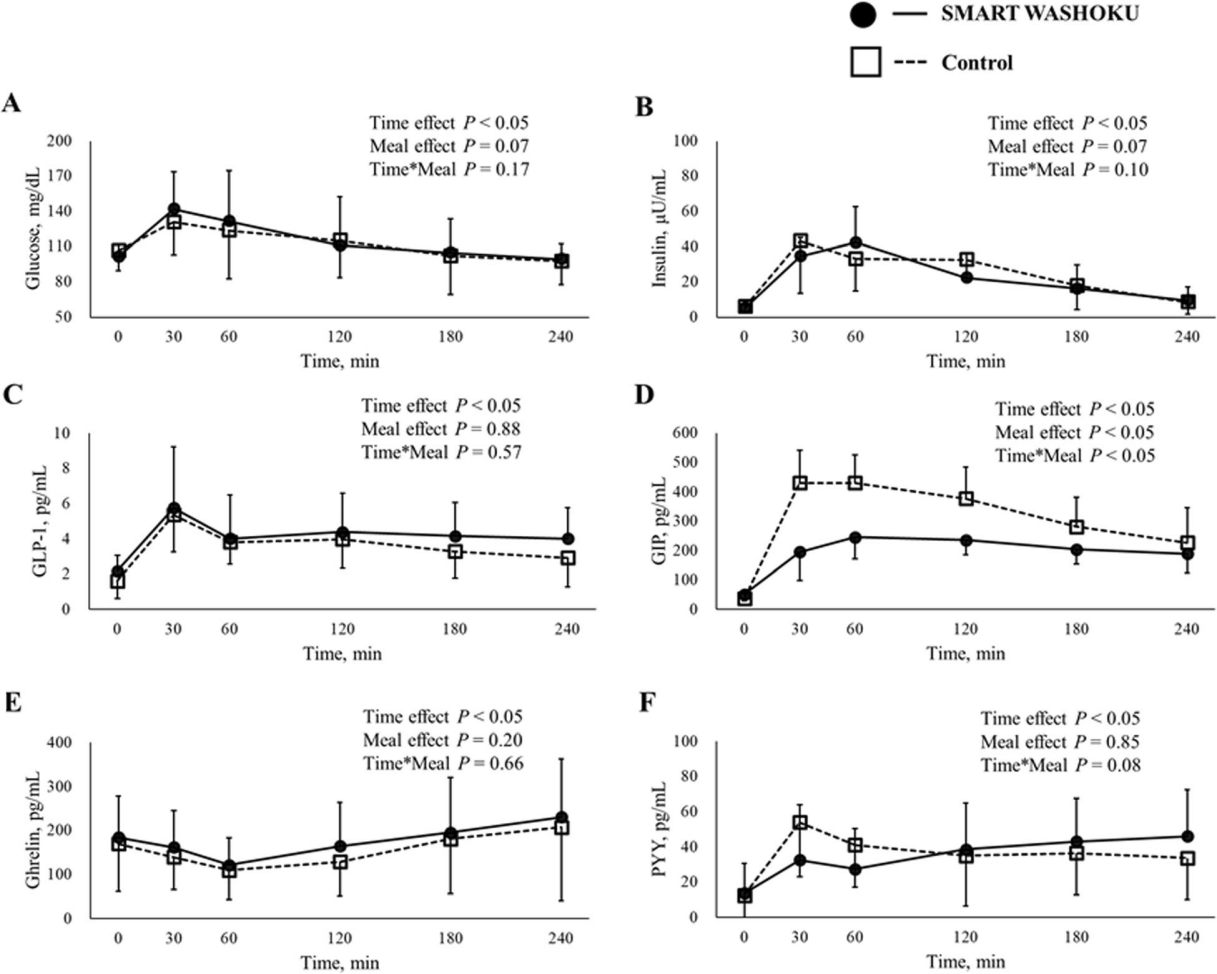
Effects of SMART WASHOKU or control meals on the secretion of glucose (**a**), insulin (**b**), GLP-1 (**c**), GIP (**d**), ghrelin (**e**), and PYY (**f**)

Participants were placed on the meal plan described earlier in the Methods section, and blood samples were collected for assessing following several variables. Relative to a control meal, SMART WASHOKU meal had a significantly lower plasma postprandial GIP concentration (AUC: 1117.0 ± 351.4 vs. 700.0 ± 208.0 pmol/L・4 h, *P* < 0.05); however, between meals, there was no significant difference in the levels of blood glucose, insulin, triglyceride, GLP-1, peptide YY (PYY), and ghrelin (Fig. 2).

### Outcomes

Relative to control meal, a SMART WASHOKU intervention produced a modest but significant reduction of VFA in our participants (− 3.4 ± 12.3 vs. -13.0 ± 9.3 cm^2^, *P* < 0.05). A carryover effect was not noted in this study (*F* value = 0.01, *P* = 0.939). Compared with the control meal, SMART WASHOKU meal did not modify the concentrations of AST, ALT, γ-GT, and hs-CRP; however, we did observe a significantly reduced in the levels of LDL-cholesterol, triglyceride, and HbA1c (Table 4). There was no difference in total energy intake between the two intervention periods. However, the protein/fat ratio, fiber/carbohydrate ratio, and n-3 fatty acids in the SMART WASHOKU meal intervention period were significantly higher compared with the control meal intervention period (Table 5).

**Table 4:** Changes in metabolic parameters following SMART WASHOKU or control meals

Variables	SMART WASHOKU	Control	*P* value
Body weight, kg	−0.97 (1.04)	−0.69 (0.82)	0.297
Body mass index, kg/m^2^	−0.33 (0.36)	−0.23 (0.28)	0.332
Body fat, %	0.05(1.30)	−0.08(1.61)	0.777
VFA, cm^2^	−13.0 (9.3)	−3.4 (12.3)	0.005
Waist circumference, cm	−1.85(1.7)	−0.86(1.6)	0.102
Hip circumference, cm	−0.17(0.9)	−0.19(0.9)	0.893
Systolic blood pressure, mmHg	−2.3(7.7)	−1.6(8.5)	0.675
Diastolic blood pressure, mmHg	−2.0(5.1)	−0.6(6.1)	0.352
Fasting plasma glucose, mg/dL	−1.7 (8.4)	−0.2 (5.2)	0.503
HbA1c, %	−0.15 (0.12)	−0.08 (0.08)	0.022
Fasting serum insulin, IU	−0.6 (1.7)	0.1 (1.9)	0.248
HOMA-IR	−0.2 (0.6)	0.0 (0.4)	0.054
HOMA-β	−3.6 (28.3)	−15.9 (64.9)	0.476
Triglyceride, mg/dL	−47.7 (57.4)	5.9 (59.4)	0.016
LDL-cholesterol, mg/dL	−25.1 (12.2)	0.95 (16.6)	< 0.001
HDL-cholesterol, mg/dL	−8.0 (4.7)	−3.1 (6.4)	0.008
AST, IU/L	0.2 (3.8)	−1.1 (4.1)	0.304
ALT, IU/L	−2.4 (8.9)	−0.2 (7.9)	0.370
γ-GT, IU/L	−8.9 (15.1)	−0.9 (8.8)	0.058
hs-CRP	0.02 (0.08)	−0.04 (0.14)	0.104

^1^ Data are presented as mean (SD). *AST* Aspartate transaminase, *ALT* Alanine transaminase, *γ-GT* γ-glutamyltranspeptidase, *Hs-CRP* High-sensitivity C-reactive protein

**Table 5:** Nutrient intake of participants between the SMART WASHOKU and control meal intervention periods

Variables	SMART WASHOKU	Control meal	*P* value
Total energy, kcal	2097 (41)	2110 (19)	0.311
Protein, %	22.7 (0.7)	13.7 (0.6)	< 0.001
Fat, %	21.0 (1.5)	37.5 (1.7)	< 0.001
Carbohydrate, %	56.3 (1.4)	48.8 (1.4)	< 0.001
Protein/fat ratio	1.089 (0.105)	0.366 (0.030)	< 0.001
Fiber/carbohydrate ratio	0.118 (0.014)	0.041 (0.005)	< 0.001
n-3 fatty acid/lipid ratio	0.125 (0.019)	0.023 (0.002)	< 0.001

Mean (standard deviation)

## Discussion

In the present study, we found that a SMART WASHOKU meal decreases VFA and serum GIP secretions. The control diet was similar to the normal diet participants ate regularly. Normal diets before intervention and during the washout period were similar and consistent with a modern Japanese modern diet [29]. It has been shown that in obese individuals, insulin resistance reduces gastric inhibitory polypeptide receptor expression and GIP activity in subcutaneous adipose tissue but not in visceral adipose tissue [30, 31]. Though GIP signaling is attenuated in the subcutaneous adipose tissue under conditions of insulin resistance, it is possible that GIP contributes to obesity by acting on visceral adipose tissue. Acute fat ingestion stimulates GIP secretion, while chronic high-fat diet loading enhances GIP secretion and induces obesity in mice [17]. Relative to the traditional diet, Japanese modern diet produced higher levels of serum GIP [32]. The Japanese traditional diet is characterized by high consumption of fish and soybean products and low consumption of animal fat and meat [9]. Thus, postprandial GIP secretion is stimulated by macronutrients, especially diet enriched with saturated fat [33–36]. In addition, fish oil, which is rich in polyunsaturated fatty acids, suppresses the postprandial GIP response more effectively than cocoa butter and olive oil [37]. However, it has been shown that in healthy young and lean females, there was no difference in postprandial GIP secretion induced by coconut fat (a source for saturated fatty acids), linseed oil, and a mixture of linseed and cod liver oil [38]. In the present study, we found that a SMART WASHOKU meal decreases serum GIP secretions during a meal tolerance test and VFA during a 2-week dietary intervention. However, these findings did not reflect the direct relationship of GIP influencing VFA. Careful attention should be paid to the interpretation of these findings. Further examination including elucidation of the mechanism is required to investigate these issues in mice and humans.

In the current study, SMART WASHOKU meal improved LDL-cholesterol levels, a result that was not observed with the control meal. This is consistent with the recent finding that GIPR antagonist inhibits the uptake of lipids and improves lipid metabolism, resulting in suppression of body-weight gain in mice [39]. Additionally, SMART WASHOKU meal resulted in an improvement in HbA1c levels and HOMA-IR. Despite a reduction in VFA and insulin resistance, the WASHOKU meal-induced mechanisms responsible for improving metabolic parameters remain unknown, warranting further investigation in this direction.

There are several reports on relationships between a low-fat diet and visceral fat reduction. Veum et al. reported no difference in the visceral fat mass after very high-fat (73% of energy fat and 10% of energy carbohydrate) and low-fat (30% of energy fat and 53% of energy carbohydrate) isocaloric diets in obese males [40]. This study was the first to show the relationships between isocaloric diets (SMART WASHOKU and modern Japanese diet) and VFA in overweight/obese males. We assigned 5 test meals for breakfast and lunch, and 5 test meals for dinner. Therefore, we suggest that it was not difficult for obese people to select foods making up SMART WASHOKU in a real-world way. In this study, decreased γ-GT due to dietary intervention was observed. Increases in γ-GT are associated with hepatic steatosis [41]. Hepatic steatosis can occur because of nonalcoholic fatty liver disease, alcoholism, chemotherapy, and metabolic, toxic, and infectious causes [42]. However, we did not conduct abdominal sonography or magnetic resonance imaging. Our dietary intervention might improve hepatic steatosis. Further examination including abdominal ultrasonography is needed to examine fat storage excess.

While the strengths of this study included a well-designed cross-over randomized trial and high-level adherence to the dietary regimen, there were also several limitations, such as the small sample size, short experimental period, and males only. Therefore, this might have limited generalizability. At least > 3-week or ≥ 12-week dietary intervention is desirable [43, 44], but long-term intervention was difficult because of the cost and burden involved. However, the two-week dietary intervention resulted in decreases in the body weight and body fat mass [45, 46]. Further examination including long-term follow-up and females is required to confirm these findings. Also, dietary adherence and intake were self-reported. Dietary adherence was self-reported. Accurate adherence may be realized by taking photos of participant’s meals. Typically, high adherence to a diet was defined as ≥80% [47]. Adherence to the dietary regimen was 96 ± 7 and 93 ± 10% for SMART WASHOKU and control meals, respectively. Taking into account the short-term intervention, we think that the rate of adherence to the dietary regimen was high. In our study, we believe that the visceral fat reduction was low because of the short duration of the experiment. However, consistent with the previous report [48], we observed that a modest reduction in VFA (− 14 to 0 cm^2^) was associated with a decrease in the number of metabolic risk factors. We adopted an Asian-specific cut-off of BMI ≥23 as overweight; therefore, the results are less generalizable for Caucasian populations.

## Conclusion

Based on our findings, we conclude that in overweight/obese men, a SMART WASHOKU meal would be helpful in decreasing VFA and improving metabolic parameters, possibly via suppressing GIP secretion. We did not measure the gut flora. Further examination including gut flora is required to investigate the association with SMART WASHOKU and gut flora in the future.

## Data Availability

The datasets used and/or analysed during the current study are available from the corresponding author on reasonable request.

## Abbreviations

AE: Adverse events
ALT: Alanine aminotransferase
AST: Aspartate aminotransferase
AUC: Area under the curve
BMI: Body mass index
CV: Coefficient of variation
ELISA: Enzyme linked immunoassay
GIP: Glucose-dependent insulinotropic peptide
GLP-1: Glucagon-like peptide 1
HbA1c: Hemoglobin A1c
HDL: High density lipoprotein
HOMA: Homeostasis model assessment
hs-CRP: high sensitivity C-reactive protein
LDL: Low density lipoprotein
PYY: Peptide YY
SD: Standard deviation
VFA: Visceral fat area
γ-GT: γ-glutamyltranspeptidase
